# A refined porcine lumbar spinal cord injury model facilitating postoperative care

**DOI:** 10.1016/j.ibneur.2026.05.004

**Published:** 2026-05-22

**Authors:** N. Prieur-Blanc, S. Trama, A. Mangini, S. Belluco, V. Callot, L. Grima, P. Decherchi, T. Marqueste, A. Hays, N. Serratrice

**Affiliations:** a Physical Medicine and Rehabilitation Center - South Hospitals, Assistance Publique - Hôpitaux de Marseille, Marseille, France; b HIPE Human Lab (UAR 202324378C - CNRS, Aix-Marseille Université, Université de Toulon & Institut Paoli-Calmettes), Marseille, France; c Anatomopathology department, VetAgro Sup, Marcy l’Étoile, France; d Aix-Marseille University, CNRS, CRMBM, Institut Marseille Imaging, Marseille, France; e AP-HM, Hôpital de la Timone, CEMEREM, Marseille, France; f Laboratory Plasticity of the Nervous and Muscular Systems,, Institute of Movement Sciences (ISM): Etienne Jules Marey (UMR 7287 - CNRS & Aix-Marseille Université), Marseille, France; g Institut Marseille Rachis, Vert Coteau Private Hospital, Marseille, France

**Keywords:** Porcine, spinal cord injury, lumbar, neuroanatomy, urinary dysfunction, functional evaluation, PTIBS

## Abstract

**Background:**

Pigs exhibit strong anatomical and neurological similarities to humans, making them valuable translational models for spinal cord injury (SCI) research. While most porcine SCI models target thoracic levels, these are often associated with significant postoperative management challenges, particularly related to urinary function.

**Methods:**

To address these limitations, we characterized a refined lumbar SCI model targeting the L3 vertebral level in pigs, based on comparative anatomy and veterinary data. SCI was induced using a contusion-compression paradigm under per-operative C-arm fluoroscopic guidance. Functional assessments were conducted at three time points: 2 days pre-injury, and 2 and 14 days post-injury. These included general activity monitoring, qualitative evaluation using the Porcine Thoracic Injury Behaviour Scale (PTIBS), and quantitative gait analysis using inertial measurement units and electro-goniometers. Animals were euthanized 3 weeks post-injury for *ex vivo* 7 T MRI and histological analyses.

**Results:**

All animals developed complete paraplegia at 2 days post-injury (PTIBS = 1), progressing to severe but partial recovery by day 14, with a significant decrease in PTIBS score (-6.5 ± 0.3) and hip extension (-36.1%). Importantly, all animals exhibited immediate and persistent urinary incontinence, avoiding the need for catheterization. MRI and histological analyses confirmed consistent and reproducible lesions at the injury site, affecting both white and grey matter.

**Conclusions:**

This refined lumbar SCI model induces reproducible motor deficits associated with urinary incontinence, facilitating postoperative management while preserving translational relevance. It provides a practical platform for investigating functional recovery and testing therapeutic strategies in SCI.

## Highlights

• Refined porcine lumbar SCI model improves postoperative management and avoids urinary retention.
• L3 contusion-compression induced reproducible paraplegia and persistent urinary incontinence.
• Functional recovery was assessed using PTIBS, IMUs, gait analysis, MRI, and histology.
• Histological and 7T MRI findings confirmed consistent dorsolateral spinal cord lesions.
• This translational model provides a practical platform for testing SCI therapeutic strategies.

## Introduction

Spinal cord injury (SCI) is a major cause of long-term disability, and the development of reliable preclinical models is essential for understanding pathophysiological mechanisms and evaluating novel therapeutic strategies. Among large animal models, pigs represent a particularly valuable translational model due to their anatomical and neurological similarities to humans, including spinal cord organization, white-to-grey matter ratio, and urinary system function (Zurita et al., 2012, Ahmed et al., 2023, Wathen et al., 2023).

In preclinical studies using porcine models, SCI is most commonly induced at the thoracic level, particularly around T10 (Weber-Levine et al., 2022, Benasson et al., 2020, Gayen et al., 2023). Thoracic injuries typically result in upper motor neuron syndromes characterized by paraplegia, spasticity, sensory deficits, and urinary dysfunction. While these models are widely used and well characterized, they are often associated with significant postoperative management challenges, especially regarding urinary retention and the need for catheterization, which may impact both animal welfare and experimental conditions.

Lumbar SCI models in pigs are less commonly investigated and are generally considered to predominantly affect lower motor neurons, leading to flaccid paralysis rather than spasticity. However, these functional characteristics remain incompletely characterized, and their implications for translational research are still debated.

Although lumbar SCI models have been previously described in pigs, their practical advantages for postoperative management, particularly regarding urinary function, remain insufficiently explored. The present study aims to provide a detailed characterization and methodological refinement of a lumbar L3 contusion model, focusing on its functional outcomes and translational relevance (Weber-Levine et al., 2022).

From an anatomical perspective, pigs typically present six to seven lumbar vertebrae depending on the breed, with the spinal cord extending caudally to approximately the S1 vertebral level before transitioning into the *cauda equina* (West et al., 2020). This anatomical configuration differs from humans, in whom the spinal cord typically terminates at L1, and may influence both lesion characteristics and functional outcomes. The L3 level in pigs corresponds to the beginning of the lumbar enlargement, a region of particular interest for modulating both motor and autonomic functions.

In addition, postoperative management and housing conditions represent critical aspects of large animal SCI models. In particular, urinary function plays a central role in animal care. From a practical standpoint, it is often easier to manage incontinent animals than animals with urinary retention requiring repeated catheterization. Based on veterinary data and comparative anatomy, particularly from canine models, an injury at the L3 level may induce paraplegia associated with urinary incontinence, thereby potentially simplifying postoperative care.

In this context, we sought to characterize a lumbar SCI model at the L3 level in pigs, using a contusion-compression paradigm, with a specific focus on functional outcomes, lesion reproducibility, and postoperative management.

## MATERIAL AND METHODS

### Animals

Four two-month-old female piglets weighing 20.98 ± 0.51 kg (*Sus domesticus,* French Landrace) each housed in a 1.75 m^2^ kept alternating between 12:12 h day (d) and night and at 21°C. The food was about 1 kg of pellets per day and water ad *libitum*. Animals were housed in the animal facility for one week in an enriched environment before the initiation of the experiment. This habituation period, during which animals regularly practiced the behavioral tasks, allowed them to decrease inter-individual differences and to reach optimal performances. The health status of the animals was controlled daily.

### Ethical considerations

Experiments conducted according to the French legislation (Decrees and orders N°2013–118 of February 1st, 2013, JORF n°0032) concerning animal care guidelines on animal experimentation, and after approval by animal Care Committees of *Aix-Marseille Université* (AMU) and *Centre National de la Recherche Scientifique* (CNRS). The authorization number granted by the French Ministry of Higher Education, Research, and Innovation (MESRI) is APAFIS #52171. All people licensed to conduct live animal experiments, and all experimental rooms have a national authorization to accommodate animals (#D1305522). Furthermore, experiments performed in accordance with the recommendations provided in the Guide for Care and Use of Laboratory Animals (U.S. Department of Health and Human Services, National Institutes of Health), with the directives 86/609/EEC and 010/63/EU of the European Parliament and of the Council of 24 November 1986 and of 22 September 2010, respectively, and with the ARRIVE (Animal Research: Reporting of *In Vivo* Experiments) guidelines.

### Anesthesia

A pre-operative fasting was performed 12 h before the procedure. On the day of SCI, piglets were premedicated in their housing box in their box with STRESNIL™ (6 cc) and ZOLETIL® 100 (8 cc) intramuscularly.

**Fig. 1:**
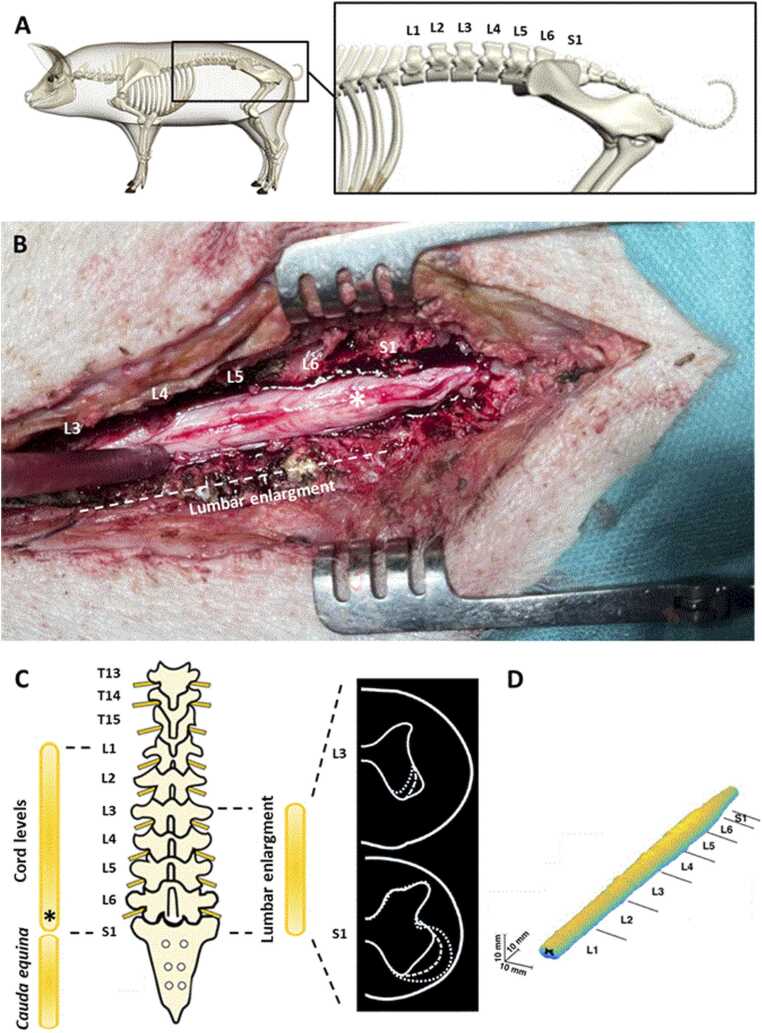
(**A**) Spine of the domestic pigs (Sus domesticus or Sus scrofa domesticus). (**B**) Anatomical picture of the spinal cord termination in pigs. (**C**) Representation of the different anatomical structures (modified from Toosi et al#, 2021) (Toossi et al#, 2021). End of the spinal cord at S1 and beginning of the Cauda equina. (**D**) 3D MRI reconstructions showing the lumbar enlargement from L3 to S1 (source?). Asterisk = Conus medullaris.

**Fig. 2:**
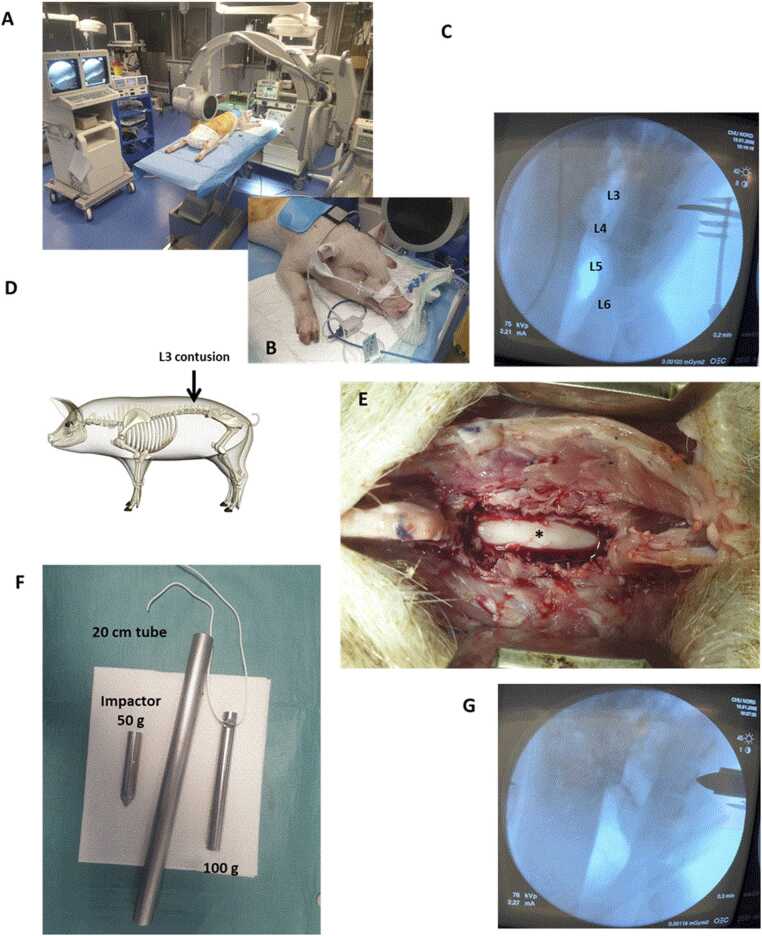
**(A & B)** Under general anesthesia, C-arm fluoroscopic identification of the L3 pedicle **(C)**. **(D)** L3 spinal cord injury. **(E)** L3 laminectomy and exposure of the *dura mater*, site of impaction (*) **(F)** Impactor (50 g weight) dropped along the rail from a height of 20 cm, a second 100 g placed in compression during 5 min. **(G)** Confirmation of correct placement of the impaction system under C-arm fluoroscopy.

A catheter was placed in the ventrolateral ear vein, and animals were intubated through the orotracheal tract. Animals were anesthetized with PROPOFOL (10 ml/h), and for pain SUFENTANYL (2 mg/h). The animal was positioned in ventral decubitus, back was shaved, disinfected (BETADINE). During surgery, body temperature was maintained at 37 °C using a homeothermic feedback-controlled heating pad. Antibiotic therapy was systematically administered 30 min before the procedure then every 1.5 h with CEFAZOLINE 22 mg/kg intravenously (Fig. 1, Fig. 2).

### SCI at L3 level under C-arm fluoroscopic control (Fig. 2)

The vertebral pedicles from L2 to L4 identified with C-arm fluoroscopy (General Electric OEC 9800). A midline dorsal incision performed over these landmarks. The muscles retracted using retractors to expose the lumbar vertebrae. After fluoroscopic confirmation of the correct level, L3 laminectomy performed to expose the *dura* without affecting the integrity of the spinal cord. To avoid any tearing of the *dura mater* during impact, a square piece of field is delicately placed in contact with it. Then, a 50 g impactor (diameter 4 mm) dropped along the rail from a height of 20 cm to strike the L3 dural sheath at the pedicle level. Immediately after impact, a second 100 g weight added onto the 50 g weight, and this total 150 g weight left in place to compress the spinal cord for 5 min. After, muscles sutured in anatomical layers, and the skin closed with separated stitches (Flexocrin 2/0). A postoperative analgesic was daily administered for 5 days (TRAMADOL 1 mg/kg every 6 h). Among the four animals, one exhibited only partial spinal cord damage due to a lateralized impact and was therefore considered as a control for descriptive comparisons. Due to the limited sample size, this control should be interpreted cautiously.

### Housing condition after the immediate surgery

In the immediate postoperative period, special care is taken with paraplegic piglets to ensure their welfare, representing an adaptation of that which is used in human clinical practice. After surgery (spinal shock phase), the animals remain in cribs all day and undergo postural changes every 3–4 h.

### Prevention of venous thrombosis

To eliminate the risk of venous thrombosis during this phase, all of our animals were given 0.04 ml of 1% low-molecular-weight sodium heparin (as prophylaxis) subcutaneously every 24 h, in the same way as is done for humans.

**Fig. 3:**
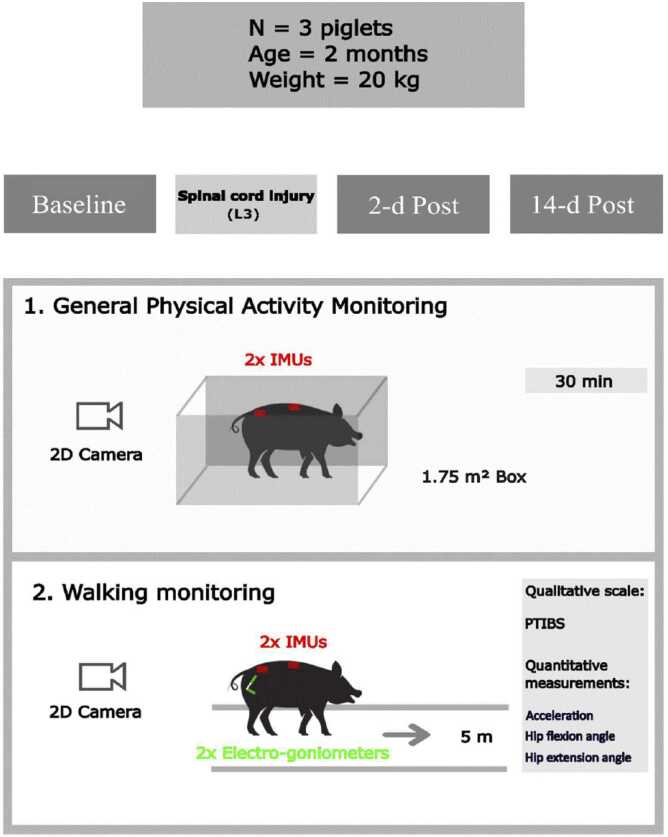
Experimental procedure of the functional piglets monitoring 2 days pre-injury (Baseline), 2 days post-injury (2-d Post), and 14 days post-injury (14-d Post). Abbreviations: IMU = Inertial Measurement Unit; PTIBS = Porcine Thoracic Injury Behaviour Scale.

### Functional evaluation (Fig. 3)

Three out of four animals were monitored at three sessions: 2-days pre-injury at the end of the acclimatisation period (Baseline), 2-days post-injury (2-d Post), and 14-days post-injury (14-d Post) to quantify (1) general physical activity, and (2) walking both (a) qualitatively and (b) quantitatively. The three measurement sessions began with the monitoring of physical activity, immediately followed by the walking monitoring.

#### General physical activity monitoring

The piglets were placed in their box (1.75 m²) and instrumented with two tri-axial inertial measurement units (IMU; Cometa WaveTrack, MI, Italy, 2 kHz) on the 4th thoracic vertebrae and the 3rd lumbar vertebrae to assess the linear and angular acceleration. The anatomical landmarks were clearly identified and checked to ensure reproducible and accurate positioning of the sensors during the measurement sessions. After a 10-min acclimatisation period, data were collected over 30 min of unsupervised activity (Qu et al., 2018). To avoid influencing the piglets' behaviour, a 2D camera (Logitech BRIO 4 K Stream Edition, 1920 × 1080 px, 60 Hz) was positioned to monitor them, but no experimenter was present in the box room.

#### Walking monitoring

For gait assessment, the piglets wore the two previous IMU in the same location and were also equipped with two bi-axial electro-goniometers (Biometrics Ltd., Gwent, UK, 1 kHz) positioned on the right and left hips. Electro-goniometers were placed to measure hip joint flexion and extension, determined by the angle between the longitudinal axis of the femur and a line that joined the *tuber sacrale* and *ischiadicum* (Jaegger et al., 2002). The electro-goniometers were calibrated in an anatomical reference position (upright and motionless piglets), to have an initial angular value of 0°, hip flexion with positive values and hip extension with negative values. Similarly to Mirkiani *et al (*Mirkiani et al., 2022*).*, the piglets were trained to follow a human experimenter along a 5-metre straight corridor, using a toy and/or food. The measurements included two crossings, with a third crossing added if the piglet failed to complete one of the previous crossings correctly. The piglets' walk was recorded in 2D using the Logitech camera, mounted on a tripod, and positioned behind the starting line.

### Functional evaluation analyses

All data analyses were performed using MATLAB software (2022a, MathWorks Inc., Novi, USA). Histological analysis and MRI revealed that three of the four monitored piglets had significant spinal cord damage, while the fourth piglet exhibited partial spinal cord damage, likely due to an excessively voluntary lateral impact. The piglet with partial spinal cord damage was retained as a CONTROL in the analyses, while the three others formed the DAMAGED group.

#### General physical activity monitoring

Raw angular acceleration was filtered using a 3rd order Savitzky-Golay filter (with a 0.051 s frame size) (Zhang et al., 2022). Similarly to the procedure of Qu *et al (*Qu et al., 2018*).*, filtered angular acceleration parallel to the dorsal plane (animal turning left or right) magnitude at thoracic and lumbar level was binned into three activity intensity levels separately: 0–5 °/s^2^ (Rest; recumbency), 5–50 °/s^2^ (Low; low-impact activities such as walking), or > 50 °/s^2^ (High; high-impact activities such as playing). Non-rest activity was calculated and defined as an angular acceleration ≥ 5 °/s^2^ and expressed in percentage of time. Non-rest activity was used as a general marker of physical activity. Recovery was calculated and defined as the non-rest activity normalised to the average Baseline value and expressed as a percentage.

#### Walking monitoring

***Qualitative scale:*** The Porcine Thoracic Injury Behaviour Scale (PTIBS) was used to assess hindlimbs function (Lee et al., 2013). Video recordings of piglets walking were viewed by three independent observers, blinded to the experimental groups (DAMAGED and CONTROL) and sessions (Baseline, 2-d Post, and 14-d Post). Walking was scored in 10 different stages, ranging from no active movement of the hindlimbs (score = 1) to normal ambulation (score = 10). PTIBS scores of 1–3 are characterised by “hind limb dragging”, scores of 4–6 reflect varying degrees of ‘‘stepping’’ ability, and scores of 7–10 reflect varying degrees of ‘‘walking’’ ability (Lee et al., 2013).

***Quantitative measurements:*** IMU’s data were resampled at 1 kHz to match the electro-goniometer. A low-pass Butterworth filter (4th order, cut-off frequency = 10 Hz) was applied to the IMU and electro-goniometer data to remove marker vibration artefacts during walking (Mirkiani et al., 2022). In order to identify when the piglets were walking, thoracic IMU was used to detect forelimbs movements, identifiable during all sessions unlike the hindlimbs. A 5-second data block was selected, and a fast Fourier transform (FFT) was applied to the medio-lateral acceleration of the thoracic IMU’s data. The FFT results were then analysed to determine the frequency range corresponding to the peak magnitude. Based on the walking speed and step time obtained in Nilsson, Pinzke, and Von Wachenfelt study (2010) **(**Nilsson et al., 2010**)**, a frequency range of 2.5–4.0 Hz was selected to identify walking periods. The IMU and electro-goniometer signals corresponding to these measurement ranges were extracted.

If a piglet managed to take at least three reciprocating gait cycles with its hindlimbs (PTIBS score > 3) during at least two measurement sessions, 10 gait cycles per session were selected to analyse gait evolution. For each gait cycle, the electro-goniometer data were interpolated to 101 points (flexion/extension along the y-axis) and the following dependant variables were calculated: maximum angle (peak hip flexion angle), minimum angle (peak hip extension angle), range of motion (ROM), gait percentage of maximum angle, and gait percentage at minimum angle. The interpolated gait cycles and their corresponding variables were averaged to obtain a single representative gait cycle per piglet and per condition. To analyse changes in gait parameters, the gait cycle variables were expressed in percentage as delta values relative to the Baseline.

### 7 T MRI procedures

Exploratory ex vivo anatomical MR explorations were conducted at 7 T on spinal cord samples to briefly describe the lesion and its extent. For that purpose, animals were sacrificed at d21 and spinal cord were extracted from L1 to S1, fixed in formol 4% with PBS, and placed in small tubes. Three out of the 4 spinal cords were scanned using a T2-weighted sequence.

### Histological procedures

After MRI, spinal cord samples were prepared for histology. For each spinal cord, 12 sections every 3 mm were prepared, dehydrated and paraffin embedded. For each obtained paraffin block, one 4 µ HE stained section was obtained, stained with hematoxylin and eosin and analyzed.

### Statistical analyses

Statistical analyses were conducted *via* R routines (R v R 4.3.2, R Core Team, 2020, R Foundation for Statistical Computing, Vienna, Austria). With regard to general physical activity and walking monitoring, we did not perform hypotheses testing or estimation procedures due to limited sample size. For descriptive purposes only, these data were described by their means ± Standard Error of the Mean (SEM).

## Results

### SCI at L3 level

With this technique, all DAMAGED animals (n = 3) were rendered paraplegic and incontinent. No animals experienced urinary retention requiring urinary catheterisation or self-catheterisation. This confirms the initial hypothesis according to which a lesion below L3 does not lead to urinary retention in pigs. A similar pattern was observed in the CONTROL piglet (lateral impact).

### General physical activity results

**Fig. 4:**
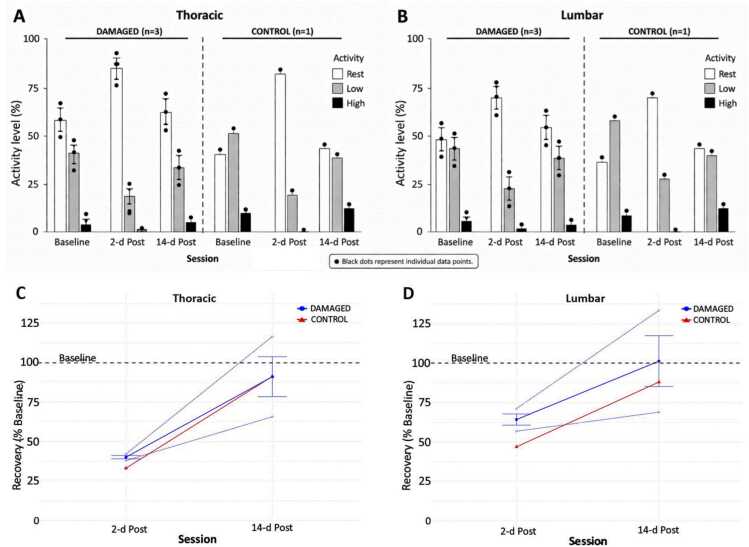
(**A**) Thoracic and (**B**) lumbar activity (rest activity in white, low activity in grey, and high activity in black) across sessions (B*aseline*: 2 days pre-injury; 2-d Post: 2 days post-injury; 14-d Post: 14 days post-injury) measured during the unsupervised monitoring. Data from DAMAGED piglets (n = 3) are represented with mean ± SEM values. (**C**) Thoracic and (**D**) lumbar recovery in percentage of Baseline for the DAMAGED group (n = 3; in blue) and for the CONTROL piglet (n = 1; in red) as across sessions (2-d post and 14-d post). Data from DAMAGED piglets are represented with unfilled blue dot (dashed blue lines) and their mean ± SEM values with blue dot filled (solid blue lines). Black dots represent individual data points for each animal (n = 3 for DAMAGED, n = 1 for CONTROL).

As shown in Fig. 4, all piglets exhibited a decrease in non-rest activity at 2-d Post compared to their Baseline values at the thoracic level (-59.8% for the DAMAGED group and −66.8% for the CONTROL piglet) and at the lumbar level (-35.6% for the DAMAGED group and −53.0% for the CONTROL piglet).

At 14-d Post, non-rest activity increased in all piglets compared to the 2-d Post session but remained lower than Baseline values at the thoracic level (-8.8% for the DAMAGED group and −8.6% for the CONTROL piglet) and at the lumbar level (-11.8% for the CONTROL piglet). The DAMAGED group had a non-rest activity 1.4% higher at the lumbar level compared to their Baseline.

### Walking results

**Fig. 5:**
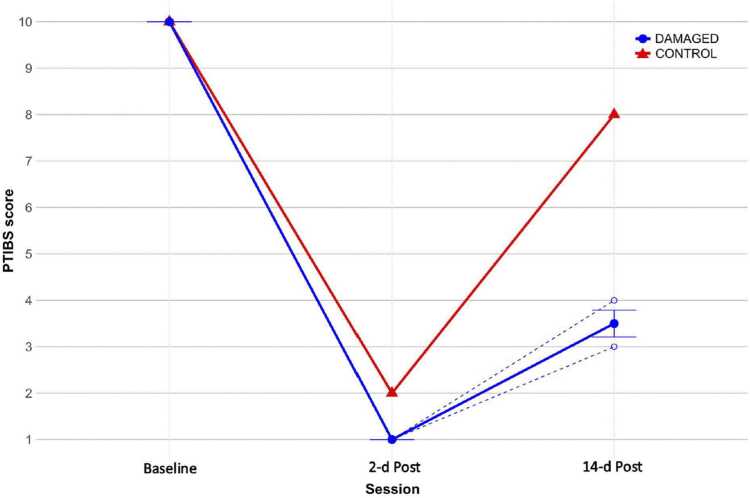
Porcine Thoracic Injury Behavioral Scale (PTIBS) score across sessions (Baseline: 2-days pre-injury; 2-d Post: 2-days post-injury; 14-d Post: 14-days post-injury) for the DAMAGED group (n = 3; dashed blue lines) and CONTROL piglet (n = 1; solid red line). Mean ± SEM values for the DAMAGED group are shown as a solid blue line.

***Qualitative scale:*** As reported in Fig. 5, all piglets demonstrated normal ambulation with normal balance and had a maximal PTIBS score (10 points) at Baseline. At 2-d Post, the DAMAGED group had no active hindlimb movements (PTIBS score of 1) and the CONTROL piglet had active hindlimb movements but, with rump and knees on the ground (PTIBS score of 2). At 14-d Post, the DAMAGED group had active hindlimb movements with “weight-bearing extensions” but were unable to take two steps with rump and knee constantly off the ground (3.5 ± 0.3 mean PTIBS score). The CONTROL piglet was able to take more than six steps with knees fully extended, but hoof placement was planter with unbalanced trunk (PTIBS score of 8).

**Fig. 6:**
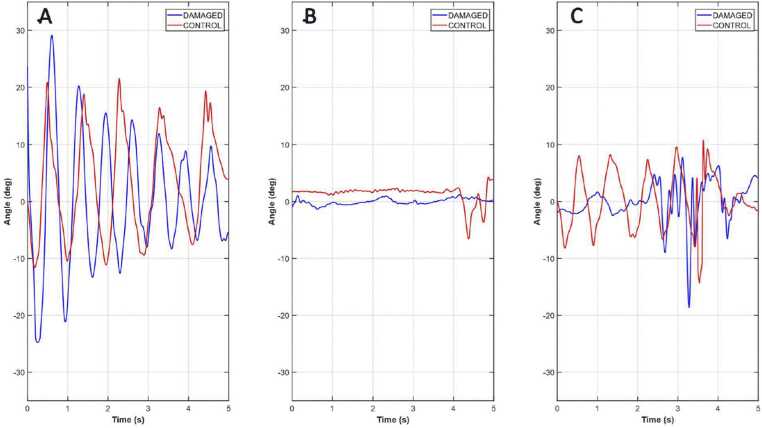
Representative hip joint angles over time during walking are shown for a DAMAGED piglet (n = 1; in blue) and the CONTROL piglet (n = 1; in red) during the (**A**) Baseline session, (**B**) 2-d Post session, and (**C**) 14-d Post session.

***Quantitative measurements:*** As shown in Fig. 6**,** in terms of hip angles, a regular gait pattern was identifiable during the Baseline session (pre-spinal impact) for both DAMAGED and CONTROL groups. This pattern disappeared at 2-d Post session after spinal cord impact, as no hindlimb gait cycle were seen via the camera or detected via the electro-goniometers. At 14-d Post session, only the CONTROL piglet and one in two DAMAGED piglets were able to take at least three consecutive steps with their hind limbs.

**Fig. 7:**
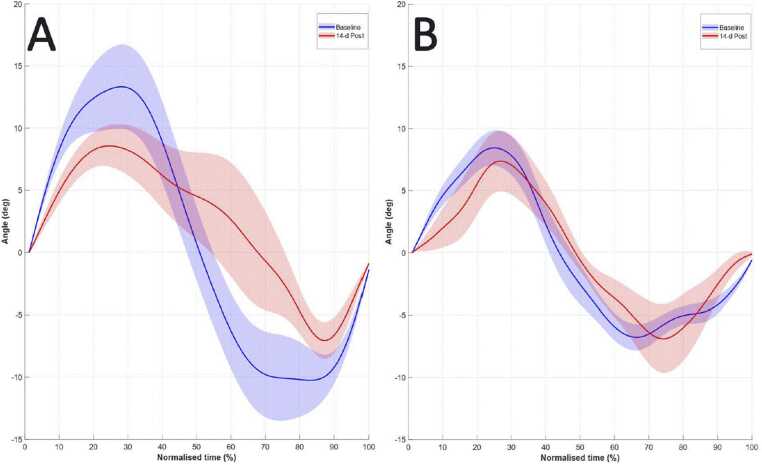
Mean ± SEM of ten gait cycles representing hip joint angles as a function of normalized time for (**A**) a DAMAGED piglet (n = 1) and (**B**) the CONTROL piglet (n = 1) during the Baseline session (in blue) and the 14-d Post session (in red).

As shown in Fig. 7, evolution of gait cycle between the Baseline and 14-d Post showed that the DAMAGED piglet had a decrease in hip flexion (-2.8%) and extension (-36.1%) resulting in a decrease in its ROM (-18.8%). Conversely, the CONTROL piglet showed an increase in hip flexion (+8.1%), extension (+33.8%), and ROM (+20.0%). At 14-d Post, maximal flexion was reached 10.0% earlier in the gait cycle for the DAMAGED piglet compared with 1.3% later for the CONTROL piglet. Minimal flexion reached 0.3% later in the gait cycle for the DAMAGED piglet compared with 8.9% earlier for the CONTROL piglet.

### Complications

#### Complications on the skin

Impaired skin integrity is among the most frequent complications seen in paraplegic animals. The risk of skin lesions (pressure ulcers) is primarily related to prolonged decubitus body pressure due to immobility. However, lesions can also be caused by friction or rubbing, especially in cribs. Another possible reason is irritation of the skin by urine or stool (incontinence ulcers). The best treatment is prevention; this can be accomplished by daily monitoring of the skin, mainly in areas of frequent occurrence of pressure ulcers, such as the sacral region, groin, knee and ankle. All the animals of this study developed local skin lesions especially on knees and ankles treated symptomatically.

#### Gastrointestinal complications

The occurrence of gastrointestinal complications in the acute phase is relatively common in this experimental model, in particular ileus. One animal had a rectal prolapse which had to be treated surgically.

#### Urinary complications

With this protocol we have no complications related to urine retention in animals. There was no urinary sepsis or renal colic for 21 days. The animals rendered incontinent did not present urinary retention events during the 3 weeks of the study, no bladder catheterization was necessary.

### 7 T MRI

**Fig. 8:**
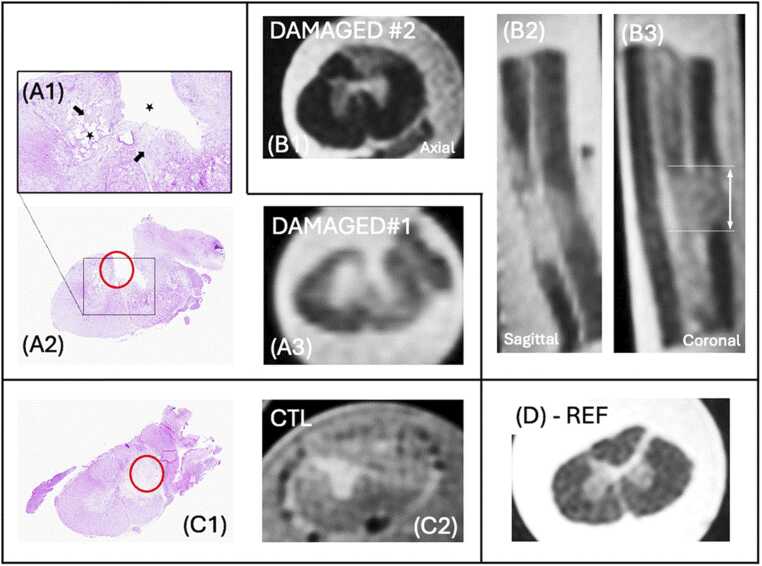
Representative histological and MRI slices collected on 2 DAMAGED piglets (**A,B**), and 1 CONTROL (**D**). (**A1**) Zoomed axial histological section collected on DAMAGED#1 showing a large lesion (star) in the dorsolateral spinal cord, which extends to the right. The observed cavity likely reflects secondary degenerative processes following the initial contusion injury rather than a direct primary mechanical effect. A large amount of Gitter cells (arrows) are visible within the cavities. A similar pattern is observed on the corresponding axial MR slice (**A3**). (**B**) MR images collected on DAMAGED#2 also show dorsolateral hyperintensities. The lesion extends over 10–14 mm (**B2,B3**). (**C**) The CONTROL piglet presented with lateral damages. (**D**) MR images collected as an additional piglet, free from SCI is provided for anatomical reference.

Spinal cord damages manifest as hyperintensities on the T2-weighted MR images. The lesion in the DAMAGED group affected primarily the dorsolateral white matter (Fig. 8 **- B1 and A3**) and, to a certain extent, gray matter (A3). The lesions extended over 5 mm (CONTROL) to 10–14 mm (DAMAGED group), as illustrated on Fig. 8 **(B2 and B3)**. On the CONTROL piglet, the lesion was located more laterally, involving one side of the grey matter and lateral tracts. A reference image (Fig. 8**D**) acquired in an additional piglet, free from injury, is provided as well, showing nice delineation of grey matter and white matter tissues.

### Histological studies

Histological images acquired in one DAMAGED and the CONTROL piglets are provided alongside the MR images in Fig. 8. The spinal cord of DAMAGED#1 presented a severe lesion (Fig. 8**-A3**), affecting 50–70% of the section at the lesion site, and extending rostro-caudally through 2–3 blocks (*i.e.* 12 mm in total). The lesion, affecting both the white and the gray matter, was dorsal in all 3 cases. Lesions consisted in a cavitation filled by a large amount of Gitter cells. Around the cavity, a moderate amount of spheroids surrounded by dilated myelin sheaths or replaced by Gitter cells (Wallerian degeneration) were visible. Within the cavity, blood vessels were present.

## Discussion

Animal models are essential for improving our understanding of spinal cord physiology and for evaluating novel therapeutic strategies following SCI (Zurita et al., 2012). Small animal models offer several advantages, including ease of handling, lower cost, and well-characterized genomes. However, their anatomical and neurological differences may limit the translation of findings to humans. In contrast, large animal models represent an important intermediate step toward clinical application. Among them, pigs share numerous anatomical and physiological similarities with humans, including spinal cord organization, white-to-grey matter ratio, metabolism, bowel function, and urinary system characteristics, making them highly relevant for translational SCI research (Ahmed et al., 2023).

Rather than proposing a fundamentally new model, our study refines existing lumbar SCI approaches by emphasizing reproducibility, functional assessment, and improved postoperative care conditions. We describe a refined lumbar SCI model, comparable to low thoracic models reported in the literature, based on a contusion-compression injury at the L3 level. This level corresponds to the beginning of the lumbar enlargement in pigs, where the spinal cord is still present—unlike in humans, where it typically ends at L1—while maintaining morphological similarities with thoracic segments. In this model, all animals developed paraplegia associated with urinary incontinence, which is easier to manage compared to thoracic models, particularly with regard to postoperative urinary care.

### Urinary consequences of an injury at the L3 level

Several considerations are critical, as the objective is to induce urinary incontinence rather than retention. Based on veterinary data and comparative anatomy, particularly from canine models, bladder control involves neural pathways originating around the L3 level. Therefore, the injury must be carefully targeted at this level to avoid upstream effects. In this context, our model induces paraplegia associated with urinary incontinence without urinary retention, thereby eliminating the need for catheterization. This finding supports our initial hypothesis that lesions at or below L3 do not result in urinary retention in pigs. From a practical perspective, this represents a significant advantage for animal care, reducing the need for invasive procedures and facilitating daily management. Moreover, urinary dysfunction remains a major clinical concern in patients with SCI, and this model provides an opportunity to monitor functional recovery in future therapeutic studies.

### Functional evaluation

The results of physical activity monitoring showed a marked decrease in non-rest activity at 2 days post-injury (≈ −63% at the thoracic level), followed by partial recovery at 14 days post-injury, approaching baseline levels (≈ −9%). Although these findings may initially appear counterintuitive, forelimb-driven movements likely contribute to the observed activity, reflecting compensatory adaptation. Similar results have been reported by Qu et al (Qu et al., 2018)., who observed a ≈ 75% reduction in activity 1 day post-injury followed by near-complete recovery at 14 days. These observations are relevant for managing secondary complications such as pressure ulcers and may also facilitate animal autonomy in feeding and drinking, thereby easing caregiver burden.

Qualitative and quantitative gait analyses confirmed normal ambulation at baseline (PTIBS score = 10) and complete paraplegia at 2 days post-injury. By 14 days post-injury, partial recovery was observed, with some animals regaining limited hindlimb function. This recovery may be explained by the contusive nature of the lesion, as well as the young age of the animals (≈ 2 months), which may enhance neuronal plasticity (Jaerve et al., 2011). The CONTROL piglet, presenting a partial lesion, showed better functional recovery, consistent with observations in thoracic models (Lee et al., 2013). Notably, substantial differences were observed between DAMAGED and CONTROL animals, including a 4.5-point difference in PTIBS score and marked differences in hip range of motion. While the CONTROL piglet exhibited a gait pattern close to baseline, DAMAGED animals primarily relied on compensatory locomotion strategies such as forelimb-driven crawling.

Histological analyses and 7 T MRI findings were consistent with functional outcomes, both in terms of lesion localization and reproducibility. This concordance supports the reliability of the model across complementary evaluation modalities.

### Limitations and perspectives

The limited sample size represents an important limitation and warrants cautious interpretation of the findings. In addition, the use of young piglets, while facilitating handling, may influence recovery outcomes due to increased plasticity (Jaerve et al., 2011). One animal in the DAMAGED group showed the ability to stand, possibly reflecting emerging spasticity, which could be further investigated in future studies. Finally, MRI and histological analyses were performed to provide a qualitative description of the lesions; more detailed quantitative analyses would be valuable to further characterize tissue damage.

This model should therefore be considered as a methodological refinement of existing porcine SCI models rather than a fundamentally novel experimental paradigm.

## ConclusionS

We describe the surgical procedure underlying a refined lumbar SCI model based on acute compression at the L3 level, along with detailed protocols for postoperative care and rehabilitation necessary to maintain paraplegic pigs in good health over several weeks.

In addition, we report the main complications associated with this model and the strategies implemented to manage them. Appropriate housing conditions, daily rehabilitation, and proactive prevention of complications—mirroring clinical care in human SCI—are essential to ensure animal survival and long-term maintenance.

Overall, this refined model is feasible, reproducible, and particularly suitable for preliminary investigations of functional recovery, including urinary function, and for testing therapeutic strategies targeting spinal cord regeneration in a translational context.

## Declaration of Competing Interest

The authors declare that they have no known competing financial interests or personal relationships that could have appeared to influence the work reported in this paper.
